# Anatomical Considerations of the Suprascapular Nerve in Rotator Cuff Repairs

**DOI:** 10.1155/2014/674179

**Published:** 2014-03-03

**Authors:** James A. Tom, Addisu Mesfin, Mitesh P. Shah, Mitra Javandel, Dan J. Lee, Douglas L. Cerynik, Nirav H. Amin

**Affiliations:** ^1^Department of Orthopaedic Surgery, Drexel University College of Medicine, Philadelphia, PA, USA; ^2^Department of Orthopaedic Surgery, Johns Hopkins School of Medicine, Baltimore, MD, USA; ^3^Southern California Orthopaedic Institute, Pomona, CA, USA

## Abstract

*Introduction*. When using the double interval slide technique for arthroscopic repair of chronic large or massive rotator cuff tears, the posterior interval release is directed toward the scapular spine until the fat pad that protects the suprascapular nerve is reached. Injury to the suprascapular nerve can occur due to the nerve's proximity to the operative field. This study aimed to identify safe margins for avoiding injury to the suprascapular nerve. *Materials and Methods*. For 20 shoulders in ten cadavers, the distance was measured from the suprascapular notch to the glenoid rim, the articular margin of the rotator cuff footprint, and the lateral border of the acromion. *Results*. From the suprascapular notch, the suprascapular nerve coursed an average of 3.42 cm to the glenoid rim, 5.34 cm to the articular margin of the rotator cuff footprint, and 6.09 cm to the lateral border of the acromion. *Conclusions*. The results of this study define a safe zone, using anatomic landmarks, to help surgeons avoid iatrogenic injury to the suprascapular nerve when employing the double interval slide technique in arthroscopic repair of the rotator cuff.

## 1. Introduction

Chronic large or massive rotator cuff tears can cause significant shoulder pain and dysfunction and may present the clinician with unique technical challenges for repair. Arthroscopic rotator cuff repair has been shown to be an effective alternative to traditional open rotator cuff repair [1, 2]. Although an increasing number of surgeons repair these types of injuries arthroscopically, mobilizing and repairing large tears using this method can be challenging, even for the experienced surgeon.

Chronic large or massive rotator cuff tears are often associated with significant muscular atrophy and retraction. Adhesions between the soft tissue and the injured rotator cuff can make the reduction of the rotator cuff tendons to bone difficult. Historically, these large tears were repaired with an open technique utilizing soft tissue releases that have been shown to be located in a safe zone that avoids injury to the suprascapular nerve [3].

Arthroscopic release of both the anterior and posterior rotator intervals, referred to as a double interval slide, is performed in order to provide adequate lateral mobilization of retracted tears for apposition to the rotator cuff footprint [4–6]. After completion of an anterior rotator interval release, a posterior interval release is directed toward the scapular spine until the fat pad that protects the suprascapular nerve is reached [3]. Injury to the suprascapular nerve may occur because of the nerve's proximity to the operative field. The purpose of this study was to define a safe zone, using bony landmarks, to avoid iatrogenic injury to the suprascapular nerve during arthroscopic repair of chronic, massive rotator cuff tears [7–9].

## 2. Materials and Methods

**Figure 1:**
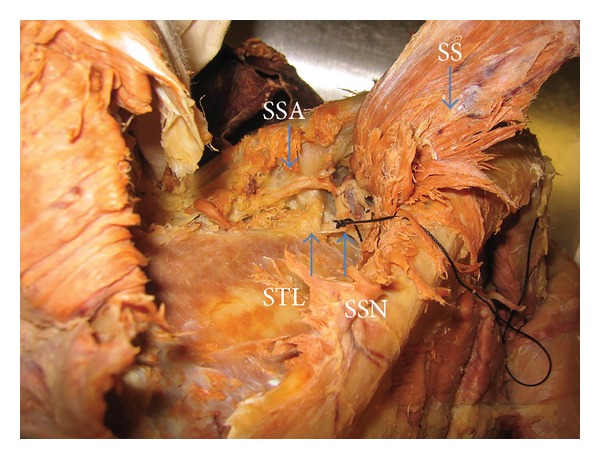
Dissection and identification of anatomical structures. SSA: suprascapular artery, SS: supraspinatus, SSN: suprascapular nerve, and STL: superior transverse ligament.

Twenty shoulders were dissected in ten formalin-embalmed cadavers (four males, six females). The average age at the time of death was 86.8 years (range: 80–96 years). None of the shoulder specimens had a history of trauma or prior surgical intervention. The specimens were placed in a prone position and a standard posterior approach to the shoulder joint was utilized. A linear incision was made from the posterolateral corner of the acromion medially along the length of the scapular spine. The skin, soft tissue, and fascia were removed. The deltoid was detached from its origin on the scapular spine from laterally to medially and reflected inferiorly, allowing for visualization of the infraspinatus muscle. The infraspinatus was retracted superiorly and the teres minor inferiorly to allow visualization of the posterior aspect of the shoulder joint capsule. The suprascapular nerve was identified and tagged as it passed inferiorly to the superior transverse ligament, and the suprascapular artery was identified and tagged as it ran over the superior transverse ligament (Figure 1). The axillary nerve and posterior circumflex humeral artery were identified and tagged as they passed through the quadrangular space. The infraspinatus muscle was then released close to its attachment on the humerus and retracted medially.

**Figure 2:**
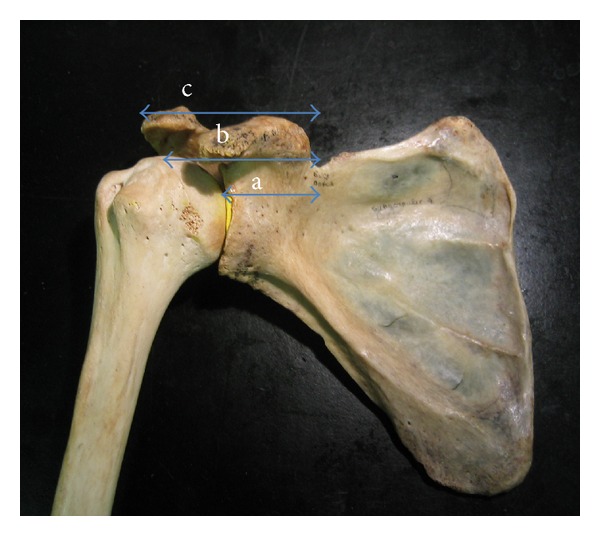
Model illustrating cadaveric anatomic landmarks for measurement. a: suprascapular notch to glenoid rim, b: suprascapular notch to the articular margin of the rotator cuff footprint, and c: suprascapular notch to the lateral border of the acromion.

With the arm in 15 degrees of abduction and neutral rotation, distances were measured from the point at which the suprascapular nerve crossed under the superior transverse ligament in the suprascapular notch to the superior aspect of the glenoid rim, the articular margin of the rotator cuff footprint, and the lateral border of the acromion (Figure 2).

Measurements were made with dial calipers (General Tools, New York, NY) graduated to a precision of 0.01 inch. The same examiner made all the measurements. Distances were measured to the nearest 0.01 inch and then converted to centimeters. Two measurements were made at each point and the average value was included for analysis. Measurements for female and male specimens were compared using a two-tailed Student's *t*-test with a significance level of *P* = 0.05.

## 3. Results

**Figure 3:**
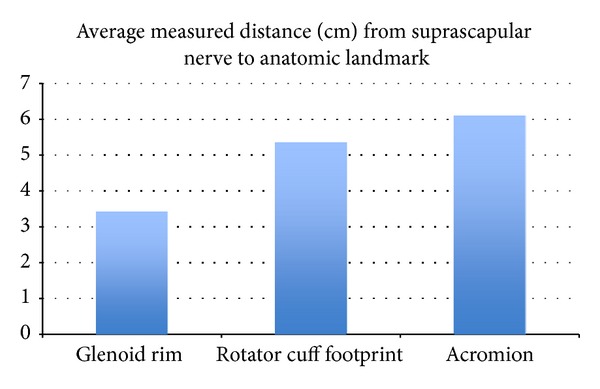
The distance in centimeters from the suprascapular notch to the chosen landmarks; GR: glenoid Rim, RCF: rotator cuff footprint, and AC: acromion.

The anatomic relationship of the suprascapular nerve to described landmarks is illustrated in Figure 3. The suprascapular nerve courses through the suprascapular notch an average of 3.42 cm (range: 3.18–3.68 cm) from the superior glenoid rim. The distance from the suprascapular notch to the articular margin of the rotator cuff footprint averaged 5.34 cm (range: 5.05–5.58 cm). The average distance from the suprascapular notch to the lateral border of the acromion was 6.09 cm (range: 5.79–6.34 cm) (Table 1).

A statistically significant difference (*P* = 0.03) was found between female and male cadavers for the distance from the suprascapular notch to the glenoid rim. The average distance was 3.36 cm (range: 3.18–3.57 cm) for females and 3.51 cm (range: 3.31–3.68 cm) for males (Table 1).

## 4. Discussion

The suprascapular nerve is a mixed motor and sensory peripheral nerve arising from the superior trunk (C5, C6) of the brachial plexus. It provides motor innervation to the supraspinatus and infraspinatus muscles as well as sensory branches to the coracohumeral and coracoacromial ligaments and glenohumeral joint.

The coracohumeral ligament is a thickening of the fibrous capsule of the glenohumeral joint and strengthens the capsule superiorly. The supraspinatus tendon is adjacent to the coracohumeral ligament and following rotator cuff tears adhesions to the coracohumeral ligament may contribute to cuff retraction and poor mobility. Releasing the soft tissues about the shoulder allows for increased mobility of the rotator cuff and eases reduction of the supraspinatus to the greater tuberosity.

The interval slide technique frees the coracohumeral ligament from the supraspinatus and allows increased mobility of the cuff during repairs of massive tears. Variations of the interval slide technique include the double interval slide and interval slide in continuity [4, 10]. During the double interval slide, an anterior release is completed, followed by a posterior interval release, which is directed toward the scapular spine between the supraspinatus and infraspinatus until the fat pad that protects the suprascapular nerve is reached.

Arthroscopic repair of chronic large or massive rotator cuff tears using a double interval slide has demonstrated promising results [11, 12]. However, overly aggressive lateral mobilization of retracted tears during release of the posterior rotator interval can place the supraspinatus nerve at risk [3]. Neurologic injuries have been reported to occur in 1–3.4% of patients undergoing arthroscopic rotator cuff repair [11, 13–15]. This number may be even higher during arthroscopic repair of massive rotator cuff tears when considering the placement of portals and suture anchors as well as the new dynamics of the repaired rotator cuff causing compression of the nerve itself [16, 17]. When performing an interval slide to lateralize the rotator cuff, it is imperative for the surgeon to understand the changes in force across the nerve.

Both the interval slide and double interval slide techniques rely on two arthroscopic portals: the lateral subacromial portal and the posterior intra-articular portal. A basket punch that is used to perform the interval release is inserted through the lateral subacromial portal, and the arthroscope is placed through the posterior intra-articular portal. A third anterolateral portal is used to perform the supraspinatus repair [5, 6].

Prior anatomic studies of the suprascapular nerve have attempted to define a safe zone to avoid injury during arthroscopic transglenoid Bankart repairs, SLAP repairs, and open surgical procedures [3, 7, 8, 18, 19]. Warner et al. evaluated the limits within which lateral mobilization of chronic massive retracted rotator cuff tears can be performed during open procedures without risking neurovascular injury. Even with delineation of these safe zones, however, iatrogenic injury to the suprascapular nerve during open rotator cuff repair and clavicle excision has been reported [16, 20].

We have described the anatomy of the suprascapular nerve as it relates to the interval slide technique. The distance from the suprascapular notch to the glenoid rim has not previously been defined and establishes a safe zone when using the basket punch in the lateral subacromial portal and the arthroscope in the posterior intra-articular portal. Knowledge of the distance from the suprascapular notch to the lateral border of the acromion may be used to establish a safe zone for anterolateral portal placement. Lastly, the distance from the suprascapular notch to the articular margin of the rotator cuff footprint is used to define the location of the nerve relative to suture anchor placement when securing the rotator cuff tendons to the greater tuberosity.

Bigliani et al. described a safe zone enabling surgeons to avoid the suprascapular nerve during arthroscopic Bankart repair and open surgical procedures. This safe zone, located in the posterior glenoid neck, measured 2 cm in diameter at the level of the supraglenoid tubercle and 1 cm in diameter at the level of the scapular spine. An average of 3.0 cm from the supraglenoid tubercle to the suprascapular notch was reported [7]. In contrast, our findings averaged 3.42 cm from the suprascapular notch to the superior rim of the glenoid.

Woolf et al. described the safety of the superior-medial portal, citing a mean distance of 2.42 cm from the suprascapular nerve [21]. While this measurement is helpful in determining a safe distance from the suprascapular nerve to this single portal, many surgeons employ multiple arthroscopic portals for rotator cuff repairs. Shishido and Kikuchi described a safe zone for avoiding suprascapular nerve injury in open dissection of the posterior shoulder joint and arthroscopic procedures for Bankart repair in which blind drilling is involved [8]. The distance reported from the suprascapular notch to the superior glenoid rim was 2.9 cm (range: 2.3–3.5 cm). This report also contrasts with our findings, particularly when considering gender differences.

To determine a “safe zone,” we took into account the normal variability of the suprascapular nerve that occurs in the setting of rotator cuff tear. As found in previous cadaveric studies, the suprascapular nerve can be translated up to 3.5 mm from its anatomic course in the presence of a rotator cuff tear [22]. To avoid potential injury to the suprascapular nerve during portal placement and soft tissue release using the double interval slide, the surgeon should maintain a distance greater than 3.86 cm from the suprascapular notch in males and 3.71 cm in females.

During repair of the supraspinatus and infraspinatus to the greater tuberosity, safe maneuvering is needed. Our study demonstrates a safe zone greater than 5.69 cm when measuring from the suprascapular notch to the rotator cuff footprint, again accommodating for anatomic variability in rotator cuff tears.

The subacromial portal is noted to be safe owing to its distance from the suprascapular nerve [6]. An estimated safe zone may be defined as greater than 6.44 cm from the nerve to the lateral border of the acromion.

Limitations of this study include the small number of shoulder specimens measured and the potential for interobserver variability when performing measurements. Additionally, variations between specimens in the location of the rotator cuff footprint, the shape of the glenoid, and the type of acromion may have affected the distances measured. Further variability may be encountered with increased specimen numbers.

## 5. Conclusion

Establishing the course of the suprascapular nerve as it relates to the operative field when employing the interval slide technique may be helpful in avoiding iatrogenic injury to the nerve. Knowledge of the relationship of the nerve to the described anatomic landmarks may help prevent iatrogenic injuries related to suture anchor and portal placement during arthroscopic rotator cuff repairs using the double interval slide technique.

## Figures and Tables

**Table 1:** The distance in centimeters* from the suprascapular notch to the chosen landmarks compared between females and males.

	GR^1^	RCF^2^	AC^3^
Female (*N* = 6)	3.36	5.36	6.05
Male (*N* = 4)	3.51	5.3	6.15
^†^	*P* = 0.03	*P* = 0.26	*P* = 0.31

^1^GR: glenoid rim, ^2^RCF: rotator cuff footprint, and ^3^AC: acromion. ^†^Two-tailed Student's *t*-test used to determine significance.

*All measurements are average values expressed in cm.
